# AI-driven radiomics and radiogenomics: supporting the assessment and differentiation of pseudoprogression in cellular immunotherapy for glioblastoma

**DOI:** 10.3389/fimmu.2026.1920605

**Published:** 2026-09-02

**Authors:** Yihua Han, Wenjing Li, Yanji Jin, Songbiao Cui, Guanghui Dong

**Affiliations:** 1 Department of Neurology, Affiliated Hospital of Yanbian University (Yanbian University Hospital), Yanji, China; 2 Department of Anaesthesiology, Affiliated Hospital of Yanbian University (Yanbian University Hospital), Yanji, China

**Keywords:** artificial intelligence, cellular immunotherapy, glioblastoma, pseudoprogression, radiomics, review

## Abstract

Glioblastoma (GBM) is the most aggressive type of primary brain tumour in adults, and after treatment, it is also difficult to know whether a person’s health has improved. Pseudoprogression (PsP), particularly following radiotherapy and combination therapy with temozolomide and new immunotherapies, may be falsely identified as true progression (TP) by conventional MRI, thus leading to premature termination of treatment or unnecessary intensification of therapy. Although RANO, iRANO and RANO 2.0 have improved the assessment of response, structural MRI alone is unable to reveal the biological complexity of the tumour microenvironment. Artificial Intelligence (AI)-based radiomics and radiogenomics aid in the characterisation of GBM. Several Magnetic Resonance Imaging (MRI) sequences are used to obtain quantitative data, such as the conventional T1-weighted images, diffusion-weighted images, perfusion-weighted images and others, to acquire information on cell density, blood vessel distribution, immune cell concentration, molecular modifications and the effect of therapy. Combine imaging characteristics with liquid biopsy, genomic data and patient health records to enhance the accuracy of diagnosis and discover high-sensitivity surrogate markers for immune checkpoint inhibitor and CAR-T cell therapy clinical trials. Clinical translation still faces numerous limitations such as inconsistent research protocols, small study cohorts, insufficient external validation, inadequate model interpretability and inconsistent reference standards. In the future, many research groups will conduct multi-centre validation, standardize workflows, open-source reporting and release clinically interpretable models. The above ways can reduce the bias induced by PsP and improve differentiation between pseudoprogression and true progression to facilitate prompt treatment for most people.

## 1 Introduction

Glioblastoma (GBM) is the most malignant grade 4 primary intracranial tumour in adults, and it has considerable molecular heterogeneity, aggressive invasion, and is inherently resistant to radiotherapy and chemotherapy (1, 2). The median overall survival and 5-year survival rate of the standard Stupp regimen are only about 14.6 months and as low as 7.2% respectively (3, 4). Due to the blood-brain barrier (BBB) and plasticity-induced resistance in rapidly evolving tumours, intensive chemoradiotherapy, antiangiogenic therapy and targeted therapy have all failed to break through this therapeutic ceiling (4). The emergence of adoptive cell transfer therapies, particularly Chimeric Antigen Receptor T-cell (CAR-T), has moved GBM treatment toward precision engineered cellular immunotherapy (5). Among the above strategies, CAR-T cells, T Cell Receptor-engineered T cell (TCR-T) cells and Natural Killer (NK) cells can be engineered to recognise tumour-associated antigens specifically (6, 7). Multi-target cell immunotherapy can be delivered locally within the brain, intrathecally or ventrally to rapidly reduce tumours and inhibit antigen escape; thus, a new treatment route for GBM has been opened.

Due to the immune-privileged status of the central nervous system, cellular immunotherapy for glioblastoma has a special diagnostic problem: immunotherapy-induced pseudoprogression (PsP) (8). PsP is fundamentally different from delayed radiation necrosis. The latter reflects late tissue injury after radiotherapy, whereas PsP represents an acute to subacute inflammatory response (9). On imaging, PsP may present as transient lesion enlargement, newly developed abnormal enhancement, and worsening peritumoural vasogenic edema, yet patients often show concurrent symptomatic improvement, and the lesions may regress spontaneously (5). Because PsP and true progression (TP) share similar imaging features, distinguishing between them remains highly challenging in clinical practice. Misclassification may lead either to premature discontinuation of an effective cellular immunotherapy or to delayed intervention when rapidly progressing tumour is mistaken for a benign treatment-related change (10).

Assessment of PsP after GBM treatment should start with a robust early postoperative MRI baseline. Contrast-enhanced MRI within 24–72 h after resection, before substantial surgery-related enhancement develops, is essential to define residual enhancing tumour and document ischemic or reactive postoperative changes (11) It also provides the key anatomical reference for interpreting later new or enlarging enhancement during radiotherapy, chemotherapy, or cellular immunotherapy. Conventional MRI is still mainly based on visual assessment, making it difficult to distinguish tumour growth from treatment-related inflammation in GBM. New imaging methods are therefore urgently needed (12). Artificial intelligence (AI)-based radiomics can capture hidden imaging features, while radiogenomics links imaging findings with molecular and immune profiles (2). This review outlines the mechanisms of PSP, discusses the limits of current assessment methods and summarizes recent advances in AI imaging to improve differentiation of PsP and true progression and assist the clinical translation of cellular immunotherapy for GBM.

Relevant studies were retrieved from PubMed, Embase, and Web of Science up to May 30, 2026. The main search strategy was as follows: (“artificial intelligence” OR “radiomics” OR “deep learning” OR “machine learning” OR “radiogenomics”) AND (“glioblastoma” OR “GBM” OR “high-grade glioma”) AND (“cellular immunotherapy” OR “CAR-T” OR “TCR-T” OR “NK cell therapy” OR “adoptive cell therapy”) AND (“pseudoprogression” OR “PsP” OR “true progression” OR “radiation necrosis”) AND (“radiomic” OR “radiogenomic”). Eligible studies focused on the application of MRI-based radiomics, radiogenomics, machine learning, deep learning, and multimodal artificial intelligence in response assessment for glioblastoma and high-grade glioma, with particular attention to their value in distinguishing PSP from true tumour progression.

This article is a narrative mini-review. The included literature was categorized and organized according to research topic and level of evidence. The review addresses the biological mechanisms of PSP, limitations of conventional imaging assessment, recent advances in AI-driven radiomics and radiogenomics, challenges in clinical translation, and future research directions. Studies were further stratified by study design and evidence level to summarize landmark findings, emerging progress, and key concepts. This approach provided a structured evidence framework to support the discussion and conclusions of each section. As a narrative review, this study summarizes major controversies, current limitations, and research gaps in the field. No quantitative meta-analysis or formal systematic evidence assessment was performed.

## 2 Biological basis of pseudoprogression in cellular immunotherapy

**Figure 1:**
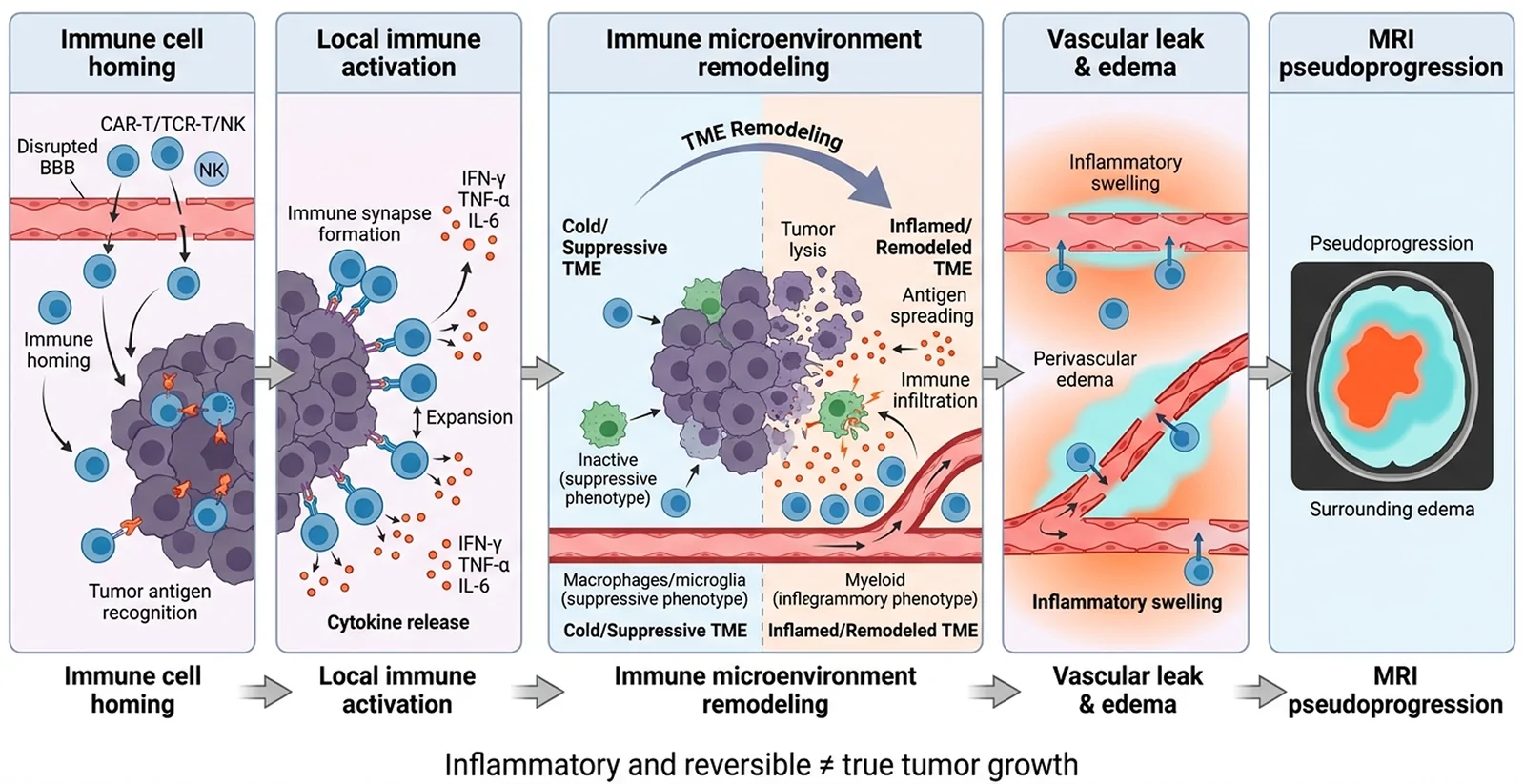
Stepwise TME remodelling cascade mediating cellular immunotherapy-associated PsP in glioblastoma (GBM): Infused CAR-T, TCR-T and NK cells cross damaged blood–brain barrier, home to tumours and bind glioma antigens. Immune synapse formation triggers lymphocyte expansion and robust secretion of IFN-γ, TNF-α and IL-6 to activate intratumoural inflammation. Tumour cell lysis releases neoantigens to induce antigen spreading, converting immune-suppressive cold TME into inflamed niche via three synergistic pathways: vascular normalization with increased permeability, pro-inflammatory polarization of tumour-associated myeloid cells, and disrupted tumour-myeloid symbiosis. Severe vascular leakage and peritumoural edema subsequently develop. These reversible inflammatory alterations manifest as transient enlarged contrast enhancement and widespread edema on gadolinium MRI, termed PsP instead of real tumour growth. Routine visual MRI evaluation fails to distinguish this inflammatory lesion from hypoxia-driven true tumour recurrence.

After local (intrathecal/intraventricular) or systemic infusion, engineered immune effector cells can home to the tumour, recognize GBM antigens, and trigger a cascade of immune responses in the tumour microenvironment (13). Their antigen-driven expansion is accompanied by proinflammatory cytokine release, which may cause central nervous system cytokine release syndrome. Meanwhile, lysis of antigen-positive tumour cells releases new antigens and activates microglia, leading to antigen spreading (13). These intense but reversible changes in the tumour microenvironment (TME) form an important basis for PsP (14). TME remodelling may drive PsP through three main mechanisms. First, therapy induces vascular normalization, which facilitates T cell infiltration by inhibiting endothelial-mesenchymal transition, restoring vascular integrity, and upregulating the expression of adhesion molecules (15, 16). Second, tumour-associated macrophages may switch from an immunosuppressive to a proinflammatory phenotype, releasing type I interferons, IFN-γ, and other mediators that increase edema and vascular permeability (17, 18). Third, CAR-T cells can disrupt tumour–myeloid cell symbiosis and cause non-antigen-specific tissue injury through bystander killing (19, 20) (**Figure 1**).

These spatiotemporally coupled inflammatory cascades can ultimately appear as transient enlargement of the enhancing lesion and a hyperperfused state. Importantly, these changes reflect reversible, immune-driven effects rather than true tumour progression. Conventional gadolinium-enhanced Magnetic Resonance Imaging (MRI) mainly captures BBB integrity and cannot reliably measure tumour burden or activity (21). True progression is largely driven by hypoxia-related abnormal angiogenesis, which causes contrast leakage and irregular enhancement, whereas PsP arises from immune-mediated remodelling of the tumour microenvironment, with inflammatory cell infiltration and disruption of vascular tight junctions leading to inflammatory exudation and enhancement leakage (21). On Fluid-Attenuated Inversion Recovery (FLAIR) images, both may show extensive hyperintensity, and without further information, it is often difficult to distinguish from infiltration of the peritumoural region by visual examination of these images alone (13, 21, 22).

## 3 Diagnostic bottlenecks in oncologic imaging

Early postoperative MRI serves as an indispensable baseline reference for differentiating PsP and TP rather than independently diagnosing PsP. It enables evaluation of resection scope, residual enhancing lesions and postoperative reactive changes within an optimal 72-hour scanning window (preferably within 48 h) (11). It helps distinguish surgical reactive changes from suspected residual tumour according to various postoperative enhancement patterns (23) and supports prognostic stratification and planning of second-look surgery (24). Nevertheless, single-session early MRI cannot reliably distinguish transient postoperative alterations from residual tumour (25), and subsequent immune-related inflammation may mimic PsP or TP. Accordingly, early postoperative MRI should be interpreted together with clinical manifestations, serial multimodal MRI, amino-acid PET and AI-assisted analytical tools to overcome these diagnostic constraints (26).

The assessment of GBM response has changed from the Macdonald criteria to Response Assessment in Neuro-Oncology (RANO), and now it also considers bidirectional enhancement, FLAIR changes, and clinical status (27). About 15%-23% of newly diagnosed GBM patients experience PsP after chemoradiotherapy, and RANO outperforms Macdonald, so it is now widely used (28, 29). RANO also has some deficiencies. The time window is fixed and sometimes inaccurate (30, 31). It gives little attention to non-enhancing lesions (32), may fail to recognise responses to new treatments such as immunotherapy (33), and is based on conventional MRI sequences with low biological specificity (34). Therefore, PsP may be overlooked or incorrectly diagnosed by about 12%-48.2% of the patients (30, 33). Immunotherapy more frequently induces PsP, thus Immunotherapy Response Assessment in Neuro-Oncology (iRANO) was proposed. If the images show progression 6 months after starting immunotherapy and there is no significant change in symptoms or neurological function, treatment will usually be continued, and an MRI will be repeated in 3 months (10, 35). iRANO also has some problems. Around 15 per cent of the cases classified as progression by RANO are labelled “unconfirmed progression” in iRANO, and in patients with actual aggressive progression, the diagnosis may be delayed by 42–93 days. Given that recurrent GBM has a median survival of only a few months, any delay will lead to extended ineffective therapy (33). RANO 2.0 has simplified and standardised the assessment, but it is still mainly based on visual examination of structural MRI. Early in immunotherapy, this is generally not sufficient to distinguish between PsP and actual progression (36).

Diffusion-weighted imaging (DWI) and dynamic susceptibility contrast perfusion-weighted imaging (DSC-PWI) are typical examples of the excellent, MRI methods that have been developed to overcome the defects of traditional structural MRI; however, each method also has its own deficiencies. DWI with Apparent Diffusion Coefficient (ADC) can quantify water diffusion, yet diffusion restriction caused by immune cell infiltration often resembles that seen with tumour proliferation, leading to a high false-positive rate (22). DSC-PWI uses relative Cerebral Blood Volume (rCBV) to estimate microvascular density, but local inflammatory hyperemia after cellular immunotherapy can also raise rCBV and mimic progression (9, 14). Even with higher thresholds or combined use of maximum rCBV and ADC, the misdiagnosis rate remains around 10% to 15% (37). Magnetic Resonance Spectroscopy (MRS) detects increased Choline/N-Acetylaspartate (Cho/NAA) as a marker of accelerated membrane turnover, but this change may reflect either active tumour growth or the release of cellular debris after immune-mediated tumour lysis, which limits its value for distinguishing true progression from PsP (37). Overall, advanced MRI mainly captures macroscopic physicochemical tissue features and still cannot specifically separate tumour progression from immune-related inflammatory change.

## 4 Artificial intelligence-driven radiomics

### 4.1 Basic workflow of radiomics in neuro-oncology

AI-driven radiomics aims to extract large numbers of quantitative features from routine images that are invisible to the human eye, then analyse them with machine learning (ML) or deep learning (DL) models (38). A typical pipeline includes multimodal imaging and biologic data collection, preprocessing and standardization, region-of-interest segmentation, feature extraction, and feature selection. In neuro-oncology, multiparametric MRI is the main data source, usually including T1-weighted (T1WI), contrast-enhanced T1-weighted (T1CE), T2-weighted (T2WI), and FLAIR sequences (39, 40). Recently, PET-radiomics has also begun to attract some attention because of the addition of metabolic information (41). Preprocessing generally includes spatial normalisation, image registration, skull stripping and intensity discretisation (42, 43). Segmentation of the tumour is necessary, but relatively demanding at this stage of the workflow (40). Given the obvious spatial heterogeneity of glioblastoma, lesions are ideally divided into subregions such as the enhancing core, the non-enhancing necrotic centre and the peritumoural oedema zone (39). Radiomic features of these areas generally include first-order statistics, texture features, shape features, and higher-order features obtained by wavelets or fractals (44, 45). Handcrafted features, such as higher-order statistical matrices, can be used to separate the disordered architecture of true progression from the inflammatory pattern of PsP. A Gray-Level Co-occurrence Matrix shows the spatial relationships of different grey levels, and a Grey-Level Run-Length Matrix counts consecutive voxels with the same intensity. Both are tissue heterogeneity (46). As many of the features are redundant, Least Absolute Shrinkage and Selection Operator (LASSO), minimum redundancy maximum relevance and embedded model-based selection methods have been employed to retain an informative subset (47, 48).

### 4.2 Construction of machine learning models for identifying pseudoprogression in GBM

Recently, ML and radiomics have been applied to extract a large number of quantitative features from conventional and advanced MRI images that are not visible to the naked eye, and built high-accuracy models based on these features. ML classifiers for identifying PsP in GBM have moved beyond proof-of-concept and are now in early clinical trials. Model performance improves when multimodal data are integrated, including radiomic features from routine T1CE and T2WI, advanced MRI sequences such as DWI and DSC or DCE perfusion, together with clinical variables such as age and molecular markers, especially O^6^-Methylguanine-Methyltransferase (MGMT) promoter methylation status (49–51). At the algorithm level, classical models such as random forest and (Support Vector Machine) SVM, deep learning architectures such as CNN-LSTM, and ensemble methods each have strengths, while hybrid models often perform better (**Supplementary Table 1**) (49–58). Still, poor generalizability remains a major limitation (59). Future work should focus on multicentre standardized data sharing and stronger external validation. Model interpretability is also essential, and tools such as SHapley Additive exPlanations (SHAP) and Local Interpretable Model-Agnostic Explanations (LIME) can help identify the features driving predictions (60). Clinical biomarkers may add value, but their deeper integration with imaging models is still incomplete. Another valuable AI application is postoperative MRI synthesis for patients without standard early postoperative MRI. Miao et al. constructed a conditional generative adversarial network, Cross-domain Correspondence Learning for Exemplar-based Image Translation (CoCosNet), to synthesize postoperative contrast-enhanced T1-weighted (T1ce) MRI using preoperative T1ce MRI and postoperative CT data of 233 glioma patients (61). Synthetic images showed good quantitative consistency with real scans (SSIM = 0.75, PSNR = 21.68, error metric = 0.007) (61). While the method cannot directly distinguish PsP and TP, it supplies baseline imaging for follow-up evaluation. When timely MRI is unobtainable, synthesized images help estimate residual lesions and ease diagnostic ambiguity in PsP/TP discrimination.

However, these performance metrics should be interpreted cautiously. Many radiomics and machine-learning studies in glioblastoma are based on relatively small cohorts, and some report only point estimates of AUC or accuracy without 95% confidence intervals, making it difficult to assess the precision and statistical uncertainty of the results. In addition, validation designs vary substantially across studies, including cross-validation, random train-test splitting, single-centre internal validation, and, less commonly, independent external validation. High AUC or accuracy values obtained from small datasets or internal validation alone may therefore overestimate real-world performance.

### 4.3 Applications of DL in AI radiomics for differentiating PsP of GBM

Handcrafted radiomics depends on predefined imaging features, and manual lesion segmentation is labour-intensive. As a result, the field is rapidly shifting toward deep learning architectures that enable end-to-end feature learning (62). Convolutional neural networks (CNN) can learn latent nonlinear spatial and temporal features directly from raw voxel data, without preset statistical matrices or manual feature extraction (Integrating deep learning and radiomics for preoperative glioma grading using multi-centre MRI data). Recent studies suggest that deep learning shows good diagnostic performance in distinguishing PsP from true progression in glioblastoma (**Supplementary Table 1**) (63–67), and in some settings matches or outperforms conventional radiomics models (67). A range of architectures has been explored, including convolutional neural networks and Vision Transformers. For example, a 3D DenseNet121 model using co-registered contrast-enhanced T1 and T2-weighted images achieved a mean accuracy of 76.4% and an Area Under Receiver Operating Characteristic Curve (AUC) of 0.756 (68). Another study reported better performance with a Vision Transformer model based on preoperative and postoperative contrast-enhanced T1 images, with an AUC of 95.2% and an accuracy of 96.7% in the validation set, outperforming a conventional CNN model (64). Nevertheless, benchmark studies have shown that although Transformer variants often achieve high AUC values, they are computationally more intensive than lightweight CNNs or Mamba + CNN hybrid models, and these latter models may offer a better trade-off between accuracy and efficiency (69). Generally speaking, the above advanced networks can acquire global whole-brain information, local lesion characteristics and changes in time from multiple follow-up imaging scans to improve diagnostic precision.

## 5 Artificial intelligence–driven radiogenomics

AI-driven radiogenomics integrates MRI phenotypes with genomic, transcriptomic and immune data to provide a non-invasive platform to support patient stratification and improve differentiation of PsP and true progression for response assessment in glioblastoma immunotherapy (70). Recently, multimodal studies have been conducted to build survival models using preoperative MRI and to correlate radiomic features with gene expression, tumour cell states and immune cell subsets; thus, it has been proposed that imaging may partially reflect the biology of the glioblastoma microenvironment (2). Baseline images can also be used to predict some molecular markers, such as MGMT promoter methylation and IDH mutations. Reported AUCs for MGMT methylation are over 0.88, and IDH prediction accuracy is more than 90% (71). Immunotherapy-related targets, such as EGFRvIII and HER2, can also be applied to radiogenomics to identify patients with suitable perfusion, diffusion and enhancement characteristics and reduce biopsy bias (71). Radiomics-pathomics models can further integrate MRI subregional heterogeneity and histopathological features to predict progression risk and help differentiate between true recurrence and treatment-related PsP (72). At the same time, multiparametric MRI combined with transcriptomic and single-cell data can be employed to track changes in CD8⁺ T-cell infiltration, macrophage polarisation and PD-1/PD-L1 or CTLA-4 expression, help detect immune resistance, and optimise the timing of combination immunotherapy (39, 70, 73). With the continuous development of AI and multi-omics integration, radiogenomics will probably be applied to personalised immunotherapy decision-making and response monitoring in GBM in the future.

## 6 Current barriers to clinical translation and multicentre promotion

Although AI-based radiomics and radiogenomics have shown encouraging but still preliminary results in single-centre studies, their wide-scale clinical application has not been realised due to data heterogeneity. Different MRI scanners, field strengths and acquisition protocols cause batch effects that reduce the stability and generalisation of the model (74). ComBat, neuroComBat and SUSAN are other ways to reduce non-biological noise and improve consistency among centres (75, 76). The Model is not transparent either. Deep learning and ensemble methods are generally less interpretable, and neuro-oncology datasets are often small; therefore, there is a risk of overfitting and limited external validation (77, 78). Current predictive models suffer from widespread incomplete methodological reporting, lacking stratified sample sizes, leakage-proof data splitting, confidence intervals, external validation and strict gold-standard confirmation. Single-centre AUC and accuracy are commonly overestimated due to small samples and inadequate external testing, limiting clinical applicability. SHAP and Gradient-weighted Class Activation Mapping (Grad-CAM) are tools that can be used to improve the interpretability of models by showing feature importance and attention maps (79). Pathological confirmation can differentiate true progression from PsP, but repeated biopsies or surgery are generally high-risk. Therefore, many studies have used follow-up outcomes as proxy labels and thus limited the training and performance of the model (62, 69). The gold standard paradox remains a major obstacle to multicentre translation of predictive models for glioblastoma PsP and TP, largely because lesion labelling criteria differ across studies (50). Histopathology from reoperation is the preferred reference standard, but it is rarely available; most cohorts depend on RANO based longitudinal follow-up, where variable imaging intervals and imperfect 2D measurements can introduce subjective bias (53, 68). Multiparametric MRI can support lesion characterization, but it still provides indirect rather than definitive evidence for PsP or TP (58). Postoperative baseline imaging is another underrecognized confounder, since models using both preoperative and postoperative contrast-enhanced T1 images perform better, whereas most studies analyse only a single follow-up scan without baseline correction (64). Labelling inconsistencies, insufficient pathological verification, and non-uniform baseline data may collectively compromise the general applicability and clinical translational potential of artificial intelligence models.

## 7 Future directions and conclusion

Future work will focus on multimodal AI that integrates MRI radiomics, liquid biopsy and clinical data to enhance the assessment of PsP (39, 80). Future PsP studies should standardize early postoperative MRI acquisition and reporting, since accurate baseline documentation of residual tumour and postoperative reactive enhancement is critical for reliable PsP/TP differentiation. AI-based MRI synthesis can serve as a practical auxiliary strategy when baseline MRI is unavailable, yet rigorous clinical validation remains essential (61). Cellular immunotherapy is now in the efficacy stage, and endpoints that only consider overall survival and structural MRI are insufficient. Validated radiomic and radiogenomic markers could be prospectively evaluated in future trials as complementary or exploratory endpoints to reduce PsP-related bias and accelerate the transformation of new therapies (70, 81). However, their clinical value should be considered provisional until robust evidence from adequately powered, patient-level, externally validated, and transparently reported studies becomes available. Future studies need full disclosure of detailed methodological and performance indicators, and pre-specified prospective multicentre validation is necessary to confirm model generalization. Despite the existing problems of standardisation, interpretability and reference norms, AI-driven radiomics and radiogenomics may support response assessment and improve the differentiation of pseudoprogression from true progression in glioblastoma, thereby providing potential support for personalised treatment.
