# Supplementary material for: AI-driven radiomics and radiogenomics: supporting the assessment and differentiation of pseudoprogression in cellular immunotherapy for glioblastoma
Source: Front Immunol. 2026 Sep 2;17:1920605. doi: 10.3389/fimmu.2026.1920605 (PMC13579400; doi:10.3389/fimmu.2026.1920605)
Supplement: Supplementary file 1 [file Table1.docx]

| **Study** | **Type of study** | | **Number of patients** | **Core data source of radiomics** | **Segmentation of ROI** | **Extraction of radiomic features** | **Feature selection methods** | **Feature variables incorporated into the model** | **Modeling method** | **Model performance** |
| --- | --- | --- | --- | --- | --- | --- | --- | --- | --- | --- |
| Lv 2024 [63] | Single-center retrospective study | | Internal training set: 10 PSP, 42 TP External validation set: 13 PSP, 17 TP | T1WI, T2WI | NA | Deep learning-derived automatic features | Network architecture adaptation | Deep learning-based imaging features | CFINet deep learning model | Internal training set:   AUC=0.929，SEN=0.976，SPE=0.860 External validation set:   AUC=0.927，SEN=0.894，SPE=0.939 |
| Li 2025 [64] | Multi-center retrospective study | | Training set: 32 PsP, 36 TuP External validation set: 8 PsP, 34 TuP | CET1 | Manually delineated | Deep learning-derived automatic features | LASSO | Deep learning-based imaging features Clinical-molecular features | CET1-ViT deep learning model | Training set: AUC=0.986 External validation set: AUC=0.993 |
| Gomaa 2025 [65] | Multi-center retrospective study | | Internal training set: 25 PSP, 34 TP External validation set: 9 PSP, 11 TP | T1 post-contrast / T1CE T2-FLAIR | Deep learning-based automatic segmentation (nnU-Net) | Deep learning-derived automatic features | SHAP | Deep learning-based imaging features Clinical-molecular features Radiation dose and temporal features | ViT Self-supervised deep learning model | Internal training set:   AUC = 0.883, SEN= 0.819, SPE= 0.720 External validation set:   AUC = 0.753, SEN= 0.727, SPE= 0.800 |
| Yadav 2024 [50] | Single-center retrospective study | | Training set: 20 PSP, 55 TP | 3D T1-MPRAGE, T2-FLAIR, post-contrast T1WI, DTI, DSC-PWI | Manual semi-automatic segmentation | Handcrafted radiomic features | RF feature importance screening | Physiological quantitative radiomic features | Machine learning (RBF kernel SVM) | Training set: AUC = 0.840, SEN= 0.7, SPE= 1 |
| Thenier-Villa 2024 [52] | Multi-center retrospective study | | Training set: 15 PSP, 15 TP | T1WI, CET1, T2WI, FLAIR, ADC | Manual semi-automatic segmentation | Handcrafted radiomic features | Principal component analysis (PCA) dimensionality reduction | Multispectral gray-scale derived radiomic features | Machine learning (MNLR) | NA |
| McKenney 2023 [51] | Single-center retrospective study | | Training set: 17 PSP, 57 TP | CET1, DCE T1 perfusion Ktrans maps, VP maps | Manually delineated | Handcrafted radiomic features | Ensemble MRMR + RFE-RF | Radiomic features MGMT methylation status | Machine learning (RFE-RF+MGMT) | Training set: AUC = 0.89, SEN= 0.72, SPE= 0.90 |
| Moassefi 2022 [68] | Single-center retrospective study | | Training set: 63 PSP, 61 TP | CET1, T2WI | Deep learning-based automatic segmentation | Deep learning-derived automatic features | Network architecture adaptation | Deep learning-based imaging features | 3D DenseNet121 deep learning model | Training set: AUC = 0.756, SEN= 0.887, SPE= 0.621 |
| Sun 2021 [53] | Single-center retrospective study | | Training set: 26 PSP, 51 TP | T1CE | Manual semi-automatic segmentation | Handcrafted radiomic features | Pearson correlation filtering + Random Forest Gini importance ranking | Top 50 radiomic texture features from T1CE | Machine learning (RF) | Training set: AUC = 0.728, SEN= 0.784, SPE= 0.613 |
| Patel 2021 [49] | Single-center retrospective study | | Training set: 30 PSP, 46 TP | CE-T1WI, T2WI, ADC | Manual semi-automatic segmentation | Handcrafted radiomic features | Pearson correlation filtering + bootstrapped cross-validated RF-RFE | Multispectral anatomical + physiological quantitative radiomic features Clinical-molecular features | Machine learning (NB) | Training set: AUC = 0.80, SEN= 0.782, SPE= 0.667 |
| Park 2021 [54] | Multi-center retrospective study | | Internal training set: 23 PSP, 63 TP External validation set: 18 PSP, 23 TP | CE-T1WI, T2WI, ADC | Manual semi-automatic segmentation | Handcrafted radiomic features | LASSO | Physiological quantitative radiomic features + Multispectral anatomical grayscale-derived radiomic features | Machine learning (SVM) | Internal training set:   AUC = 0.90, SEN= 0.783, SPE= 0.829 External validation set:   AUC = 0.80, SEN= 0.667, SPE= 0.870 |
| Park 2020 [55] | Multi-center retrospective study | | Internal training set: 17 PSP, 36 TP External validation set: 20 PSP, 13 TP | CE-T1WI, ADC, nCBV perfusion map | Deep learning-based automatic segmentation Manual semi-automatic segmentation | Handcrafted radiomic features | Embedded feature selection based on random forest | Multispectral anatomical + physiological quantitative radiomic features | DLAS model Machine learning (RF) | Internal validation set: DLAS model AUC = 0.81, accuracy = 86.8% Manual segmentation model AUC = 0.81, accuracy = 86.8% External validation set: DLAS model AUC = 0.78, accuracy = 63.6% Manual segmentation model AUC = 0.65, accuracy = 54.5% |
| Li 2019 [66] | Single-center retrospective study | | Training set: 23 PSP, 61 TP | Single-modality DTI grayscale MRI images | NA | Deep learning-derived automatic features | Network architecture adaptation | Deep learning-based imaging features | DC-AL GAN deep learning model | Training set: AUC = 0.947, SEN= 0.976, SPE= 0.883 |
| Akbari 2020 [56] | Multi-center retrospective study | | Internal training set: 10 PSP, 35 TP External validation set: 10 PSP, 10 TP | Multispectral anatomical sequences, DTI quantitative maps, DSC perfusion maps | Manually delineated | Handcrafted radiomic features Deep learning-derived automatic features | Sequential feature selection No independent screening algorithm adopted | Multispectral anatomical + physiological quantitative radiomic features Deep learning-based imaging features | Machine learning (SVM) | LOOCV:  PSP: AUC = 0.919, SEN = 80.0%, SPE = 88.68% TP: AUC = 0.835, SEN = 80.0%, SPE = 89.29% Hold-out:  PSP: AUC=0.842, SEN=75.00%, SPE=89.47% TP: AUC=0.803, SEN=83.33%, SPE=72.73% |
| Elshafeey 2019 [57] | Multi-center retrospective study | | Training set: 22 PSP, 76 TP | DCE-derived Ktrans permeability map DSC-derived leakage-corrected rCBV perfusion map | Manual semi-automatic segmentation | Handcrafted radiomic features | MRMR | Physiological quantitative radiomic features | Machine learning (SVM) | Training set: AUC = 0.891, SEN= 0.914, SPE= 0.882 |
| Jang 2018 [67] | Multi-center retrospective study | | Internal training set: 22 PSP, 37 TP External validation set: 8 PSP, 11 TP | Gd-enhanced T1-MPRAGE MRI | NA | Deep learning-derived automatic features | Deep image features: automatically selected via CNN-LSTM Clinical features: RF | Deep learning-based imaging features Clinical-molecular features | CNN-LSTM deep learning model | Training set: AUC = 0.83 |
| Booth 2017 [58] | Single-center retrospective study | | Training set: 12 PSP, 38 TP External validation set: 7 | Conventional axial T2-weighted spin-echo MRI | Manually delineated | Handcrafted radiomic features | Unpaired t-test filtering Random Forest feature importance ranking | Multispectral gray-scale derived radiomic features | Machine learning (SVM) | LOOCV: AUC=0.9, Accuracy 88% Hold-out: Accuracy 86% |
| **Comparison** | | | | | | | | | | |
| Lv 2024 [63] | | Compared with VGG, ResNet, DenseNet and existing cross-modal interaction networks such as MMTM, CDMAF and CMFI, CFINet achieves the optimal performance among all models. | | | | | | | | |
| Li 2025 [64] | | 1. The CET1-ViT model with ViT-B/16 backbone outperforms mainstream CNNs including VGG16, ResNet50, DenseNet121, Inception_v3 and MobileNet_v2 in discrimination performance. 2. Multimodal models integrating clinical molecular features with CET1-ViT deep imaging features achieve further significant performance improvement. | | | | | | | | |
| Gomaa 2025 [65] | | 1. This self-supervised multimodal ViT deep learning model outperforms mainstream existing models such as random forest, CNN-LSTM and CNN-SVM. 2. Multimodal fusion models combining MRI images, clinical indicators and radiotherapy dose features achieve better performance than models built solely on imaging or clinical features alone. | | | | | | | | |
| Yadav 2024 [50] | | SVM with RBF kernel achieves superior overall performance compared with other conventional machine learning models, including KNN, LR, linear SVM, RF and MNN. | | | | | | | | |
| McKenney 2023 [51] | | Models constructed with dual feature screening via Ensemble MRMR combined with RFE-RF outperform imaging-only models and models built on all raw features without pre-filtering. | | | | | | | | |
| Sun 2021 [53] | | The machine learning model yields better diagnostic performance than three radiologists with visual reading alone. | | | | | | | | |
| Patel 2021 [49] | | 1. The combined clinical-radiomic model achieves the optimal performance, significantly outperforming models based on single feature sets. 2. Machine learning models integrating radiomic, clinical and molecular features possess far stronger diagnostic power than radiologists’ visual evaluation alone. | | | | | | | | |
| Park 2021 [54] | | 1. The machine learning model built on SVM outperforms AdaBoost and KNN. 2. The ADC map-based radiomic SVM model achieves the best cross-center generalization performance. | | | | | | | | |
| Park 2020 [55] | | 1. DLAS-based radiomic models match semi-manual segmentation for distinguishing post-chemoradiotherapy GBM true/pseudoprogression and generalize better externally. 2. Manual delineation takes 15–20 min per case; DLAS auto-segmentation takes only 20 s, reducing labor and inter-observer bias. | | | | | | | | |
| Li 2019 [66] | | DC-AL GAN-based model significantly outperforms DCGAN, VGG, ResNet and DenseNet. | | | | | | | | |
| Akbari 2020 [56] | | The interpretable handcrafted APS radiomic features achieved optimal overall performance with stable generalization. | | | | | | | | |
| Elshafeey 2019 [57] | | Models built with linear kernel SVM outperform C5.0 decision tree in both predictive efficacy and interpretability. | | | | | | | | |
| Jang 2018 [67] | | 1. CNN-LSTM deep learning architecture extracting temporal imaging features outperforms conventional machine learning (RF) significantly. 2. The model achieves optimal performance when integrating imaging and multi-dimensional clinical data. | | | | | | | | |

Supplement Table 1. AI-Driven Radiomics for Diagnosing Pseudoprogression of GBM
